# Eighth Report to the Legislature of Massachusetts, Relating to the Registry and Returns of Births, Marriages and Deaths, in the Commonwealth

**Published:** 1852-02

**Authors:** 


Art. VI.—*Eighth Report to the Legislature of Massachusetts*, relating to the registry and returns of births, marriages and deaths, in the Com monwealth. By Amasa Walker, Secretary of the Commonwealth. Boston. Dutton & Wentworth. 1851.

As stated in a prefatory note by Mr. Walker, this report—a very elaborate and interesting document---was drawn up by Josiah Curtis, M. D., to whom we are obliged for a copy of it. It makes a volume, neatly and tastefully executed, of one hundred and thirty large octavo pages, more than half of which are statistical tables, and of which we propose to give as complete an analysis as we can do in the time and space at our command.

The report covers twenty months; from the 1st of May, 1848, to December 31st, 1849; and embraces an analysis of thirty-eight thousand three hundred and thirteen *births;* ten thousand nine hundred and fifty-one *marriages;* and thirty thousand five hundred and ninety-five *deaths.*

In Table 1, is given the *births* in Massachusetts during eight months of the year 1848, from which it appears that the greatest number occurred in August, and the next greatest in September, whilst the smallest number occurred in May. The same fact is exhibited in tables giving the births in 1849; and is probably to be explained by the circumstance, that most marriages are contracted in November, and that the close of autumn is the season when food is most abundant and the average of general health is highest. It is in that month, or the one succeeding, that, for these reasons, conception ought oftenest to take place. Cases of twins in 1848, were most numerous in July, and next to that in August; but in 1849, the plurality cases were most frequent in September and October, attaining the highest number in the latter month. In January, the number of these cases was fifteen; in February, seventeen; while in September they were thirty-six, and in October forty-nine.

"Each annual return," says Dr. Curtis, "shows that a majority of those born in the State are males, while a majority of those who die are females." The proportion of births in 1849, was 52 to 48 per cent., and of deaths 49.53 to 50.47, showing a greater difference between the sexes in the births than the deaths. In reference to *marriages*, the following results are brought out by the tables:

"A remarkable uniformity will be noticed to exist generally in the above tables, between the proportions which the several months furnish in the one year and the six years. The months, however, differ widely from each other. November indicates that more than twice as many marriages have been solemnized in that month as have taken plnce in either of the several other months. February, March, July and August do not differ much in their proportion, and they are found to furnish less than any other months of the year.

Age presents also quite an interesting topic for consideration. During the twenty months we find marriages among persons of all ages intervening 13 and 91. The youngest individual married was a female of 13 years, several instances of which occurred. The youngest male was 16, who married a female of 19; the youngest couple was a male of 17 and a female of 14; a male of 20 and another of 25 married each a female of 13; a male of 19, one of 21, aud another of 27, married each a female of 14; two males of 25 each, two of 28 each, one of 30, one of 35, and another of 47, married each a female of 15; and a bachelor of 50 married a girl of 19.

Although the male was usually the eldest of the allied couple, yet many instances happened where the reverse obtained; thus we find a male under 20 married a female over 40; a bachelor of 24 married a widow of 42; a bachelor under 35 married a widow over 60; and another bachelor under 40 married a widow over 75. A female of 18 married the *second* time, and one of 59 married the *fifth* time. A male of 30 married the *third* time. One of 36 and another of 45 married the *fourth* time, each. Among those at later ages in life, we find a male of 81 married a female of 69; but the oldest couple married were Mr. Calvin Kilborn, of Princeton, and Mrs. Susan Saunders, at the respective and respectable ages of 91 and 70. He is a farmer in good health, of sprightly habits and good mental faculties, still remembering the scenes and "incidents of travel" which he experienced in 1777, when he went as fifer at the Bennington Alarm. It seems worthy of notice that in this office, and almost side by side, are the official records of Mr. Kilborn's enlistment in Capt. John White's company which marched to Bennington in July, 1777, and also of his marriage in November, 1848, more than three score and ten years having intervened the interesting events. He has always been able to do the work on his farm to the present time, with but little assistance."

As to the probabilities of marriage, it appears that under the age of twenty years, they are on the side of females, fifteen to one; that between the ages of 20 and 25, they are much nearer equal, though still somewhat in favor of the female; but that after the age of 25, till death, they are about two to one in favor of the male. Dr Curtis continues:

"Again we perceive above, that of all females married, the chances that this interesting event will take place prior to the age of 20, are about as *one to four* of all the probabilities that they will ever marry; that is, when a female arrives at the age of 20 years and is unmarried, nearly *three quarters* of her probabilities are lost, and if she is unmarried at the age of 30, she has passed nearly *nine-tenths* of her chances of ever becoming a wife. The case is different with males, more than one half of whose marriages occur subsequent to the age of 25. But the period of life between 20 and 25 appears the most probable of all the quinquennial periods for matrimonial alliances to both sexes."

The mortality of the State was signally increased, in 1849, by the prevalence of cholera, 20,423 deaths having occurred in that year against 12,934 in 1848. This epidemic exhibited in the cities and towns of Massachusetts the same preference for foreigners that has been manifested by the disease in so many other places in our country, and was strikingly shown in this city last summer. "Of the 707" says the Report, "who had it in Boston, 572 were foreigners, 460 of these being natives of Ireland; and only 135 were born in this country, 42 having been born in Boston, and 42 more in other parts of this State."

Of *Dysentery* the Report, after mentioning that the mortality from it was very great, goes on to remark, that,

"In its late visitations it differs as much, or more, from the ordinary type of that disease, as cholera differs from cholera morbus, or as typhus does from the plague of former times in older countries. The years 1848 and 1849 were particularly noted by the terrible mortality from this destroyer. For six years previous to 1847, the annual average of deaths from dysentery was only 236, but in 1847 it rose to 1,074, and during the succeeding twenty months, which are embraced in this Report, it numbered 4,590, of which 2,455 were in 1849. By far the most fatal year was 1848, immediately preceding the visit of the cholera. In the latter eight months of that year, 3,135, or nearly a fourth part (23.53 per cent.) of all deaths in the State, occurred from it, besides those in the city of Boston. In 1847 and 1848, this epidemic was principally confined to cities in the eastern part of the

State, but in 1849 it appears to have chosen more rural localities, 67.53 per cent. being in the country districts, and only 32.47 per cent. in cities."

Typhus fever appears to have diminished in the State, but measles, scarlet fever, and small pox have increased within the last seven years. The latter disease was the cause of one hundred and fourteen deaths, in 1849, and of only one hundred and six, during the whole of the seven years previous to 1848. Diseases of the digestive organs, says Dr. C., "have also constantly increased of late." But the great outlet to human life in Massachusetts is phthisis pulmonalis.

"Of the 102,596 cases of death recorded in this State (including the city of Boston) within the last nine years, and whose diseases were specified, (besides those who died by violence,) 22,342 are stated to have been from consumption of the lungs! that is, a little more than one out of every five deaths, (21.78 per cent.) This may be confidently stated as very near the general law, or degree of relationship, which this formidable terminator of human life bears to the aggregate of all other diseases in this Commonwealth. Besides these from consumption, 4.88 per cent. of all deaths, of known diseases, were from inflammation of the lungs, making 26.66 per cent., or somewhat more than one quarter of the mortality from these two common diseases of the lungs alone!"

The influence of *season* upon health is shown in statistical tables, from which it appears that the summer months in that northern latitude, as well as in regions farther south, "are by far the most fatal."

"August and September present an aggregate of 30.70 per cent., which is more than double the amount shown by any other two contiguous months, and nearly double the amount of any two selected months. The two months above named show a difference from each other of only the half of one per cent. in the State; but in the divisions of city and country it is much greater, and stands against August in the former, and against September in the latter. In some of these cities, isolated, it is still greater; being in the city of Boston, for instance, 19.94 per cent. in August, and only 10.88 per cent. in September. Previous to 1840, September was the most fatal month in the mortality of Boston, and prior to 1820 it was in October that the most deaths occurred. This change has taken place gradative, as Boston has become more densely populated, and more insalubrious to its residents, particularly the younger portion of them. In the country districts, the abstracts show us that September is at this time the most fatal of either of the twelve months. Had time permitted us to have made the requisite abstracts from the records, it would most probably have been demonstrated to us that the excessive mortality of summer in cities, especially, was, to a very unproportioned degree, sustained by children. Of all who died in the city of Boston, in the months of August and September, 1849, omitting the deaths from cholera, which mostly prevailed among adults, we find that 56.24 per cent. were under five years of age.

Among the diseases which seem to prefer particular seasons of the year, we observe generally that zymotics and those which are severe upon tender life, mostly select the hot weather of advanced summer. Dysentery in its epidemic type, as it has prevailed of late, seems particularly to be almost confined to a short period, while the arch disease, consumption, runs through all seasons with nearly equal results."

Under the head of *Occupations* not many statistics are given, but the few presented are significant and instructive.

"Thus, of *agriculturists* or *farmers*, we find nearly five thousand (4974) with an average age of 63.83 years. Of ordinary *laborers* there were 2282, many of whom were probably foreigners, with less healthy habitations than the home of the planter. With the laborer we find the average age to be only 45.39, being 18.44 years less than the average life of the husbandman. A similar disparity is noticed also, in examining the number of each of those classes which were furnished by the separate years.

Again, let us compare the two trades, *carpenters*, who are not confined by their labors to one place, or to in-door influences, and the *shoemaker*, who is subject, under present arrangements in most workshops, to serious influences, tending to deteriorate health and abridge life. The 662 carpenters lived an average age of 49.28 years, while the 1011 shoemakers enjoyed an average life of only 43.04 years, being 6.24 years less than their more fortunate brethren just alluded to, and 20.79 years less than the highly favored farmer. This difference, though quite sufficiently important to arrest attention, receives additional claims to notice, when we consider that the race which finds a goal at such unequal distances, does not commence prior to the 21st year of life. Taking the extreme cases, we find the farmer and the shoemaker, at the age 20, with a prospect of living 43.83 years extended to the former, while that of the latter is curtailed to only 23.04 years, showing a dlfference of nearly one hundred per cent. Laying aside all considerations more elevated than those of merely a pecuniary element, and we find the farmer paying the same premium for life or health assurance as those of other callings in life, although the latter may have no chance of living much more than one half as long as the former."

While the mortality in the State, during the years 1848 and 1849 was over two per cent., "from an accumulation of facts," says Dr. Curtis, "it is evident that it might be reduced and kept as low as 1.50, or at least 1.75 per cent.;" and if thus reduced, more than four thousand lives would have been saved to the State in each of those years. These deaths may therefore be set down as having occurred unnecessarily; and the conclusion is, that much yet remains to be done even in that well-ordered Commonwealth, "for the preservation and general welfare, physically, socially, morally, and pecuniarily, of its citizens." Let us add, that nothing is so well calculated to turn public attention to these improvements, and to bring about the needed reforms, as these registration reports. We cannot believe that abominations such as are detailed in the following extract, which we make from the report of Dr. Curtis, can long appeal for abatement to an enlightened and Christian community:

"Of Lowell, says Rev. Mr. Wood, the philanthropic and humane minister at large of that city, 'There is one infraction of the laws of life and health among the poor, which, I think, should be brought more prominently before the public. This is in connection with their habitations. Their rooms are generally not ventilated at all. From six to ten persons frequently sleep in a single room, and sometimes in one bed. . . . Cellars are occupied in very damp locations, where water frequently stands in drops on the walls, and in wet times can be wrung from the sheets of the bed. Two-thirds of the inhabitants of the city, probably, would not deposit their vegetables where not a few families reside. I know of one case, where, in two connected rooms in a cellar—and lighted by only three small panes of green glass covered with cobwebs, and where, on entering, I stumbled over the beds, because I could not see them—four families, amounting to twenty-two souls, were living!' A late dispensary physician of the same city, writes: 'Very few are aware of the hundreds of places now inhabited by a horde in a horrid condition. One week ago I entered a house in a central location, and found it occupied by one store and twenty-five different families, embracing one hundred and twenty persons, more than half of whom were adults! In one of the rooms, which was inhabited by two families, I found one of the families to consist of a man, his wife, eight children, (four of whom were over fifteen years of age,) and four adult boarders!'

Of Boston, Dr. Buckingham, late dispensary physician, writes: 'I have seen the tide pouring into a back yard, from all four sides, to the depth of a foot; and have known men to sail around their kitchens in pursuit of their dinners, and coast along the shores of their cellars in tubs, for their winter's wood!' In the City Physician's Report on the Cholera in Boston, in 1849, we find that 'one cellar was reported to the police, to be occupied nightly, as a sleeping apartment, by thirty-nine persons! In another, the tide had arisen so high that it was necessary to approach the bedside of a patient, by means of a plank, which was placed from one stool to another, while the dead body of an infant was actually *sailing* about the room in its coffin!" '

